# Towards Low-Cost Effective and Homogeneous Thermal Activation of Shape Memory Polymers

**DOI:** 10.3390/ma6125447

**Published:** 2013-11-27

**Authors:** Andrés Díaz Lantada, María Ángeles Santamaría Rebollo

**Affiliations:** Product Development Laboratory, Mechanical & Manufacturing Engineering Department, Universidad Politécnica de Madrid, c/José Gutiérrez Abascal 2, Madrid 28006, Spain; E-Mail: santamaria.marian@gmail.com

**Keywords:** shape-memory polymers, electrotextiles, conductive paint, conductive polymers, smart materials and structures

## Abstract

A typical limitation of intelligent devices based on the use of shape-memory polymers as actuators is linked to the widespread use of distributed heating resistors, via Joule effect, as activation method, which involves several relevant issues needing attention, such as: (a) Final device size is importantly increased due to the additional space required for the resistances; (b) the use of resistances limits materials’ strength and the obtained devices are normally weaker; (c) the activation process through heating resistances is not homogeneous, thus leading to important temperature differences among the polymeric structure and to undesirable thermal gradients and stresses, also limiting the application fields of shape-memory polymers. In our present work we describe interesting activation alternatives, based on coating shape-memory polymers with different kinds of conductive materials, including textiles, conductive threads and conductive paint, which stand out for their easy, rapid and very cheap implementation. Distributed heating and homogeneous activation can be achieved in several of the alternatives studied and the technical results are comparable to those obtained by using advanced shape-memory nanocomposites, which have to deal with complex synthesis, processing and security aspects. Different combinations of shape memory epoxy resin with several coating electrotextiles, conductive films and paints are prepared, simulated with the help of thermal finite element method based resources and characterized using infrared thermography for validating the simulations and overall design process. A final application linked to an active catheter pincer is detailed and the advantages of using distributed heating instead of conventional resistors are discussed.

## 1. Introduction

Shape memory polymers are active materials that present a mechanical response to external stimuli; usually changes in temperature. Although other stimuli such as light or chemicals may promote shape memory effects in polymers, we focus here on thermally activated shape memory polymers as they are the most common ones. When these materials are heated above their “activation” temperature (*T*_act_), a radical change takes place from rigid polymer to an elastic state, which in some cases allows deformations of up to 400%. After being manipulated, if the material is cooled with the imposed deformation, this structure is “frozen” and returns to a rigid but “unbalanced” state. When the material is once again heated above its “activation temperature” (normally corresponding to glass transitions or melting point) it returns to its initial non-deformed state. The cycle can be repeated numerous times without any degradation to the polymer and most suppliers can formulate different materials with activation temperatures of between −30 °C and 260 °C, according to the application required. Among the polymers developed that possess shape memory, the most important are epoxy resins, polyurethane resins, cross-linked polyethylene, diverse styrene-butadiene copolymers, and other formulations described before [1,2,3,4].

They are, therefore, active materials that possess thermomechanical coupling and an ability to recover from high deformations (much greater than that of shape memory metal alloys), which combined with their lower density and cost has encouraged the design of numerous applications. Their properties permit applications in the manufacture of sensing devices or actuators, particularly for the aeronautic, automobile and medical industries. The recent proposals for their medical use have been examined previously [5,6]. However, if the development of new and more demanding applications is to be encouraged, especially for the medical industry, and if implantable devices for human beings are to be obtained, the synthesis, processing, modeling, prototyping, characterization and environmental response of these materials need to be given a very close examination [1,2,4,7,8,9,10,11].

Another limitation of intelligent devices based on the use of shape-memory polymers as actuators is linked to the widespread use of “punctual” distributed heating resistances, working via Joule effect heating of small resistors connected in series, as activation method [6,10]. Joule effect heating using resistors involves several relevant issues needing attention, such as: (a) Final device size is importantly increased due to the additional place required for the resistances; (b) the use of resistances limits materials’ strength and the obtained devices are normally weaker; (c) the activation process through heating resistances is not homogeneous, thus leading to important temperature differences among the polymeric structure and to undesirable thermal gradients and related stresses, also limiting the application fields of shape-memory polymers.

In relation to the progressive improvement of shape memory polymer capabilities and optimization of their activation process (searching for alternatives to punctual heating resistances), it is worth mentioning the use of nickel nanoparticles, carbon black, carbon nanotubes (amongst others), embedded inside the material, in order to obtain electroactive shape memory polymers whose heat-based activation process is faster, more controllable and more efficient, as a result of the homogeneous distribution of the heating particles. Additional information on electroactive shape memory polymers can be found consulting [12,13] for explaining the use of carbon nanoparticles [14,15], for a description on using nickel nanoparticles and [16] for a specific review on the topic. More recently, pioneer research [17,18] describes the use of nanopapers with embedded nanotubes, as coating for promoting the conductivity of shape-memory polymers and enabling their activation by heat transfer from the nanopaper to the shape-memory polymers, which opens new activation possibilities, highly linked to the alternatives presented in our current study.

It is important to remark that in previous devices based on shape memory polymers, typically activated using heating resistances (as well as in recent studies from our group using Peltier heater-coolers [19]), temperature differences around 30–60 °C can usually be found within the core of the polymer, while these novel electroactive shape memory polymers provide temperature differences normally lower than 30 °C among the whole structure. Such homogeneous and more controlled behavior has potential for enabling medical applications, as the references explain in depth. Another interesting possibility is linked to the incorporation of micro and nanoparticles into shape memory polymeric devices or structures for promoting induction heating, thus achieving remote activation of the shape memory effect, with notable prospects in terms of the development of active implantable devices, as wireless devices and implants can be thus developed [20,21]. However, the impact of nanoparticle inclusion on the mechanical properties is also relevant and should be addressed, as well as the influence of processing on final device cost.

It is important to note that these aforementioned solutions, linked mostly to the incorporation of nanoparticles into the polymeric matrix or to the use of nanocomposites, require systematic synthesizing and processing methods, equipments not always available, as well as special security issues linked to working with nanoparticles. In our present work we describe other interesting alternatives, based on coating shape-memory polymers with different kinds of conductive materials, including textiles, polymeric films and conductive paints, which stand out for their easy, rapid and very cheap implementation. Distributed heating and homogeneous activation can also be achieved in several of the alternatives studied and the technical results are comparable to those obtained by using advanced shape-memory nanocomposites. The following section describes the materials and methods used, before presenting and discussing main results of current research.

## 2. Materials and Methods

### 2.1. Shape Memory Epoxy and Activation Materials

Proof of concept probes and application rapid prototypes have been obtained via additive laser stereolithography using a shape memory epoxy sold under the trade name of Accura^®^ 60 (3D Systems, 333 Three D Systems Circle, Rock Hill, SC 29730, USA) the properties of which are listed in Table 1. In order to obtain more detailed information about material properties, a supplier’s data sheets can be consulted or they can be directly contacted for more specific questions. Additionally, some studies have shown the utility of carrying out dynamic mechanical analysis for obtaining full knowledge about the properties of parts manufactured by laser stereolithography. That research has used photocurable epoxy resins, similar to the one employed in our trials, for evaluating the effects of different influencing factors, such as part geometries, machine precision, processing conditions, post-cure time or the inclusion of different additives and reinforcement fibres [22,23,24,25].

**Table 1:** Properties of the materials used for prototype manufacture.

Accura^®^ 60 epoxy resin from 3D systems	
Property	Value
Density	1.21 g/cm^3^
Tensile strength	58–68 MPa
Young’s modulus	2690–3100 MPa
Activation temperature (*T*_act_)	58–62 °C
Hardness-Shore D scale	86

It would be also important to study in depth how several aspects can modify the properties of these materials, especially their activation temperature. For example, environmental humidity or physical ageing have proven to be of importance and should be carefully taken into account, as previous research has shown for different polymers [9,26]. We would also like to note that very recent advances have led to the development of new photosensitive materials for stereolithography, especially focused on the medical industry, whose shape memory properties should be thoroughly analysed, in order to promote the design of active implantable medical devices [27,28,29]. Anyway, the Accura^®^ 60 epoxy resin used has helped us to study the possibilities of activating shape memory polymer structures and devices, by heating through low-cost coatings based on electrotextiles, conductive threads and paints.

The different coating materials have been acquired from Mindsets Online (The IO Centre, Lea Road, Waltham Cross, Herts, UK, http://www.mindsetsonline.co.uk) and include: (a) a kit of electrotextiles with samples of “electronylon”, “nickel electronylon”, “clearmesh”, “softmesh”, “electrolycra” and “steel-cloth”; (b) a roll of conductive thread including Ag nanoparticles and (c) a sample of conductive ink with distributed carbon nanoparticles.

Additional information about the properties and components of the different electrotextiles can be found by consulting the data sheets from the manufacturer and obtained by personalized characterization, as we have done for some of the properties needed for the FEM-based thermal simulations (see below). In short, the different electrotextiles are woven from metallic threads or using conventional cloth threads covered or embedded with metallic particles. A remarkable issue is the low cost of the different coatings used (for all the samples used in present study we invested less than 20 €), especially when compared with other interesting solutions, such as the use of nanoparticles for changing the properties of shape memory polymers, which would have led to an investment around 10 times higher. The coating process is also direct, as detailed a couple of subsections below.

### 2.2. Computer-Aided Designs and FEM-Based Thermal Simulations

Several computer-aided design and engineering (CAD-CAE) programs allow for the application of finite-element modeling (FEM) for simulating thermo-electro-mechanical phenomena during the development process of a novel product. In our case, the different geometries of the probes (50 × 10 × 2 mm^3^) and of the final application device (active catheter end) were designed with the help of NX-8.5 (Siemens PLM Solutions), whose advance simulation “NX-Nastran Thermal” module has been also used for the FEM-based thermal simulations of the different configurations under study. We studied four different configurations, according to the main possible combinations and geometrical distributions of the epoxy probes and the related coating (for thermal activation) materials, so as to analyze and select the most adequate ones for the final validation with real prototypes. The configurations include: (a) one-side coated probe; (b) two-side coated probe; (c) coating enclosed within two half-thick probes and (d) thread distributed upon probe. Detailed description of the materials, meshes, thermal loads, boundary conditions, solver parameters and post-processing options are included under these lines.

(1) Material and mesh:

The command “3D swept mesh” has been used for meshing the epoxy probes, with an element size of 0.5 mm, and for meshing the coatings, with an element size of 0.085 mm. The mechanical and thermal properties of the epoxy resin were incorporated to the program, according to the values of Table 1 and Table 2.

**Table 2:** Additional properties and data important for thermal simulations.

Property	Value
Thermal conductivity (epoxy)	0.2 W/(m·K) at 25 °C
Specific heat (epoxy)	1200 J/(kg·K)
Emissivity (epoxy)	0.86
Convection coefficient	20 W/(m^2^·K)
Room temperature	298 K

(2) Loads and boundary conditions:

Increasing thermal loads were applied to the geometries of the conductive coatings, for the four different configurations, until the steady-state temperature fields in the epoxy probes reached values above the activation temperature, in this case 60 °C. Values of 1–1.2 W applied to the coatings of configurations “a”, “c” and “d” led to the desired activation temperature, while a value of 0.5 W applied to each coating of configuration “b” would also promote activation, according to simulation results. Previous experiences from our team with shape memory devices of similar material and overall mass also required heating powers near to 1 W [10]. Perfect surface contact between the coatings and the epoxy has been supposed and its implications will be further discussed, when analyzing the resemblance between simulation results and thermal images upon real prototypes in the discussion section.

A convection coefficient of 20 W/(m^2^·K) has been applied to the outer surfaces of the different configurations and geometries, according to typical values for free air convection [30] and to results from previous trials in our Product Development Laboratory [10]. An emissivity of 0.86 for the epoxy resin has been taken into account, a parameter which has been also considered when carrying out the trials with physical prototypes supported by infrared thermography.

(3) Solver parameters and post-processing:

From the different possibilities of NX-8.5 for carrying out FEM simulations, NX-Nastran solver and thermal analysis are selected with the option of “element iterative solver” activated as 3D elements are used for the simulations. Simulations are carried out at a default ambient temperature of 25 °C. Once the simulations are carried out, post-processing tools allow for an easy measurement of temperature fields and heat flows.

Both steady-state and transient simulations were carried out for the different configurations, so as to obtain information about the heating power (via Joule effect) needed to reach the activation temperature of the material and to assess the overall evolution of temperatures during the heating process, as well as the time needed to activate the probes and appliances.

### 2.3. Prototypes and Trials

**Figure 1:**
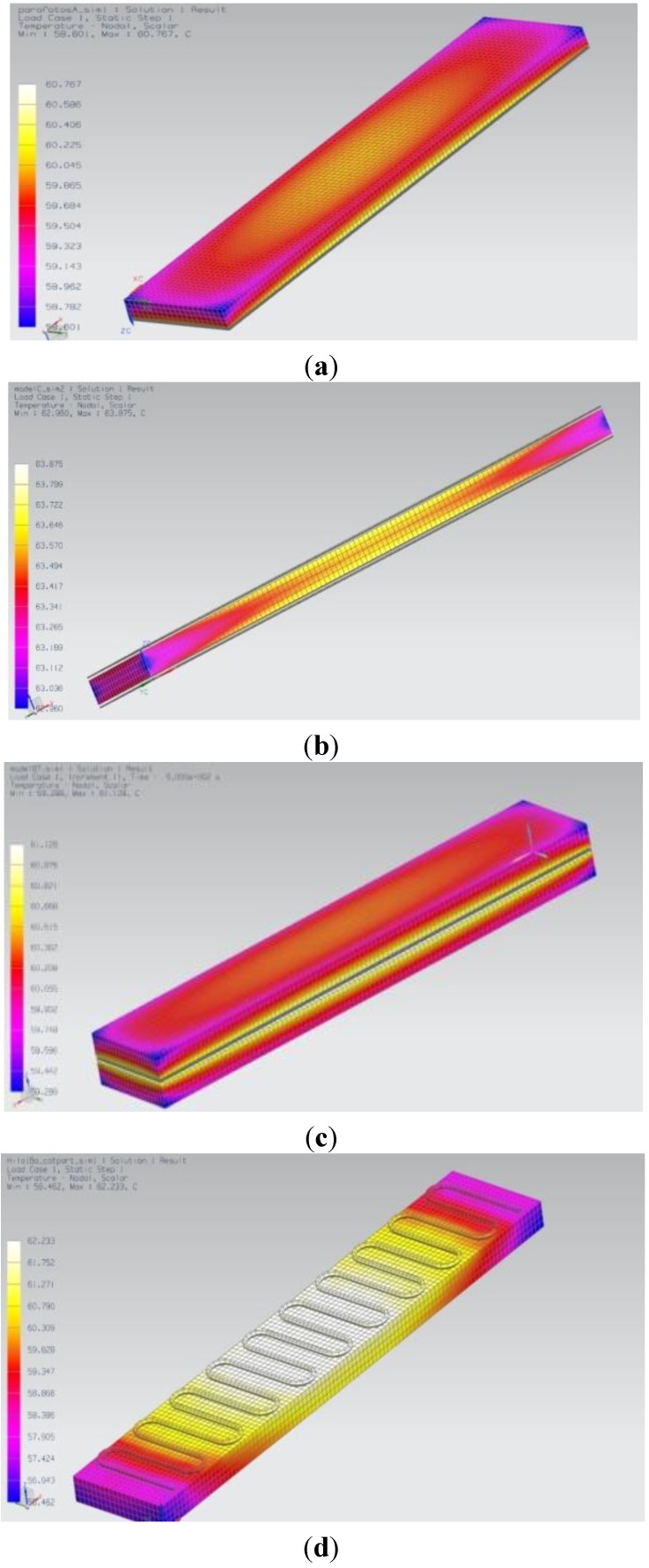
Results from thermal FEM simulations (steady-state solution): Temperature fields obtained for the different configurations, when heating beyond activation temperature.

Simulation results (see following section and results from Figure 1) led us to the manufacture of probes and final appliance coated according to configurations “a” and “d”, due to their simplicity and adequate homogeneous results. Rapid epoxy prototypes have been manufactured using our SLA-3500 laser stereolithography machine from 3D Systems, capable of reading the information about part geometries from the original CAD files and subsequently manufacturing them using a layer-by-layer approach. The possibility of using laser stereolithography and the typical shape memory properties of epoxy resins has been previously highlighted as a rapid way of conceptually validating intelligent devices based on these stimuli responsive materials [31].

**Figure 2:**
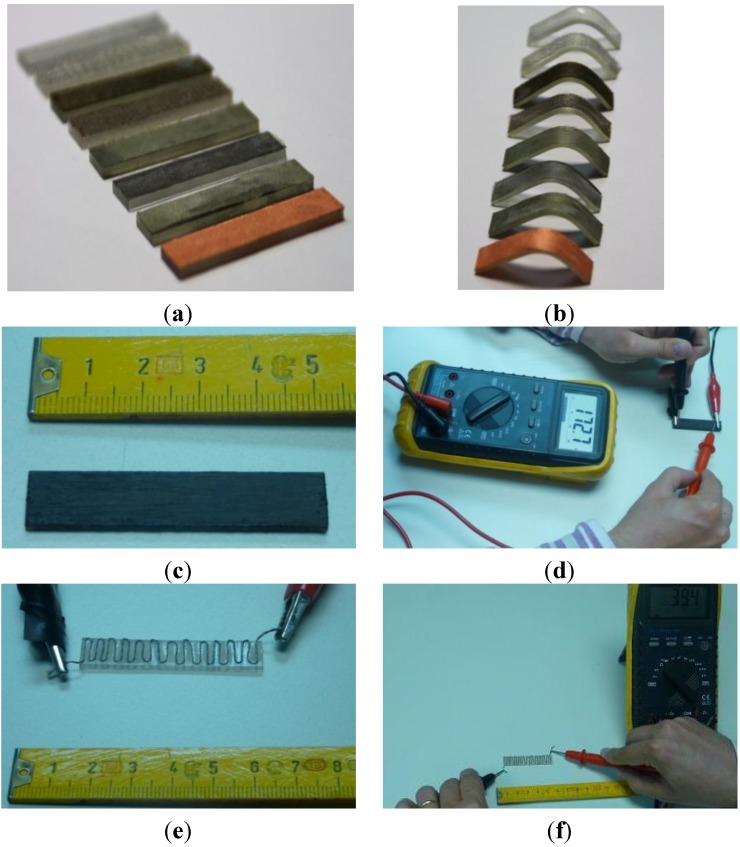
Examples of (**a**) different probes coated with different electrotextiles; and (**b**) temporary geometries obtained after training of the shape memory effect. Examples of conductivity measurements upon (**c**,**d**) different probes coated with conductive ink; and (**e**,**f**) thread.

Once the prototypes are obtained, the different coatings (electrotextiles, conductive thread and ink) were either glued or applied to one of the surfaces of the probes or devices (see Figure 2). Electrical resistivity, between the extremes of the different probes, has been measured using a Mastech MY-68 digital multimeter with a measuring range of 0.1–50 MΩ, as shown in Figure 2, and main values obtained are included in Table 3.

Some electrotextiles, woven using just metallic threads, led to very low values of resistivity. However, the textiles using conventional cotton or polymeric threads embedded or coated with metallic particles, as well as the conductive ink, led to more interesting values according to our previous experience [32], as the present trials have also shown. Regarding the conductive ink, it is important to mention that increasing the number of applied (painted) layers helps to decrease the value of electrical resistivity, so it can be tuned by the user, and that such value changes importantly during the drying process.

In our case, one application of conductive ink led to a resistivity of 260 Ω/cm and two applications led to a resistivity of 127 Ω/cm, while this value further decreased after complete drying to a final 35 Ω/cm, we believe due to shrinking after elimination of water content, which promotes contact between the conductive carbon particles.

In any case, it is relevant to measure such values and prepare the trials considering them, together with the thermal FEM simulation results, so as to avoid short-circuits and in order apply the adequate voltages for the desired thermal loading.

**Table 3:** Electrical resistivity of the different coatings.

Electrical resistance measured between the extremes of the probes	Value
Electronylon	≈0 Ω/cm
Nickel electronylon	≈0 Ω/cm
Clearmesh	≈0.2 Ω/cm
Softmesh	4 Ω/cm
Electrolycra	10 Ω/cm
Steel cloth	0.1 Ω/cm
Conductive thread	0.4 Ω/cm
Conductive ink	35 Ω/cm

The training process of the shape-memory effect has been carried out by heating the probes, with the coatings already applied, in a convection oven at 80 °C and by forcing them against a rounded border until the extremes of the probes form an angle of 90°. Maintaining the deformation they were cooled down to room temperature. Some examples of the temporary geometries attained can be seen in Figure 2.

The application prototypes, with the geometry of an active catheter end or pincer, have been also trained by deformation at high temperature and subsequent cooling down. One of the prototypes has been trained to a temporary geometry with the two sides of the pincer forming an angle of 90° and the other one has been deformed until the two sides form an angle of 160°. In that case the conductive ink is applied after the training process.

Shape recovery trials are carried out by connecting the extremes of the different probes to a variable voltage source. According to simulation results, heating powers of around 1W in the one-side coating configuration should lead to the adequate temperature rise, so as to reach the activation temperature and promote the geometrical recovery. For each probe, the voltage is selected according to the electrical resistance previously measured (Table 3) by use of Joule’s law (*P* = 1 W = *V*^2^/*R*, being *V* the voltage applied, R the electrical resistance and P the heating power). For the probes with very low resistances it is advisable to connect in series some additional resistances or a variable resistance, forming a voltage divider, so as to avoid short-circuits. In the case of the active pincer, the voltage is also connected to the extremes using a couple of “crocodile” clamps.

Infrared thermography tools have already proved their usefulness for designing, testing and characterizing shape memory polymer-based devices, improving both control over the trials, results assessment and overall process security [32]. They were also used once again, as an aid to testing the devices produced, for controlling the temperature at every instant in the different zones of the prototypes and for easily following the geometric changes, once the activation temperature of the zones of interest is exceeded. We used a “Flyr Systems Thermacam E300”, together with the analysis software “Thermacam Reporter 8.0”. Main results from simulations and trials are included and discussed in the following section.

## 3. Results and Discussion

### 3.1. Results from Thermal Simulations: Analysis of the Different Configurations

Figure 1 shows results from the thermal FEM simulations, for the steady-state solution, in the form of color maps of the different temperature fields obtained for the four configurations, when heating beyond activation temperature. The results are also summarized in Table 4, including the heating powers applied to each coating of the different configurations and the steady-state temperature range (minimal and maximal temperatures in permanent regime) obtained in the simulations.

**Table 4:** Summary of thermal simulation results for the different configurations.

Configuration	Heating power applied to each coating	Steady-state temperature range
(a) One-side coating	1 W	[58.6–60.8 °C]
(b) Two-side coating	0.5 W	[59.1–59.8 °C]
(c) Coating sandwiched between two half probes	1.2 W	[59.2–61.1 °C]
(d) Thread upon probe	1.2 W	[56.4–62.2 °C]

A relevant aspect is that the use of conductive coatings as heating elements, in all the configurations, led to much more homogeneous steady-state temperature fields than the use of punctual heating resistances, according to the data of previous simulations and trials using such punctual heating elements [10,32]. Regarding the different configurations, the two-side coating led to the most uniform temperature, while the thread glued upon the probe led to some higher differences, although the temperature variation range of 6 °C is well below most common experiences with conventional resistors.

The results from the thermal simulations helped us to select the most appropriate configurations for constructing the prototypes. As configurations “a”, “b” and “c” led to very similar results; we opted for the one-side coating, due to its simplicity (Figure 2a–d). For the prototypes using the conductive thread as heating element the only possibility is the “thread upon probe” (Figure 2e,f), although some improvements towards more homogeneous heating can in this case be achieved by surrounding a squared section probe with a conductive thread, as discussed in following sections.

### 3.2. Results from Shape-Memory Effect Activation Trials

**Figure 3:**
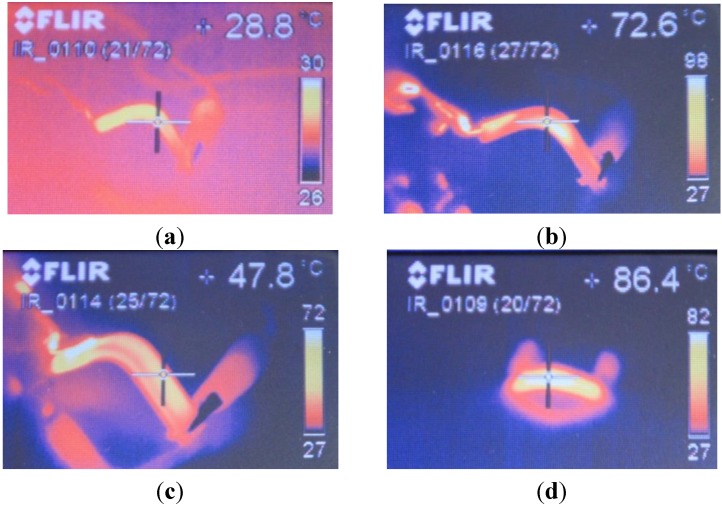
Infrared thermographs as examples of some heating processes and the related shape recoveries, as well as some difficulties, with different probes coated with electrotextiles. Commercial denominations of the different electrotextile coatings shown in the thermographs: (**a**) “electronylon”; (**b**) “clearmesh”; (**c**) “steelcloth”; (**d**) “softmesh”.

Figure 2 includes some examples of the different probes coated with different electrotextiles and of the temporary geometries obtained after training of the shape memory effect. Examples of conductivity measurements upon different probes coated with conductive thread and ink are also included as final part of the preparation before the shape recovery trials, through Joule effect heating by the current passing through the coatings. Figure 3 includes infrared thermographs as examples of some heating processes and the related shape recoveries, as well as some difficulties, with different probes coated with electrotextiles, as detailed further on.

Some of the electrotextiles used (both electronylons) have such low values of electrical resistivity that the application of a voltage between the extremes of the probes would lead to short-circuits. On the other hand, if electrical resistances are connected in series, both for security and for more controlled evaluations, these coatings act as a prolongation of the circuit coils and almost no heating can be appreciated. The major part of these probes reached temperatures around 30 °C (with results similar to those shown in Figure 3a), well below the activation temperature, so these coatings can be discarded for the activation of shape memory polymers.

Other electrotextiles also led to problematic results (clearmesh, electrolycra and steel cloth), as they consist of metallic threads woven in parallel and perpendicular patterns. Therefore, when a voltage is applied, main current flows along the few parallel threads, accounting for an effective resistance much lower than measured and promoting very relevant temperature differences among the major part of the probes, almost at room temperature, and the over-demanded threads which end up burning (Figure 3b,c).

Among the different electrotextiles, from the kit purchased, the best results are obtained with the softmesh (Figure 3d), which led to a homogeneous heating and an adequate shape recovery. In addition, such electrotextile is very flexible and does not mechanically affect the training and recovery processes, while more conventional solutions based on resistors or inductive coils dramatically do [10].

**Figure 4:**
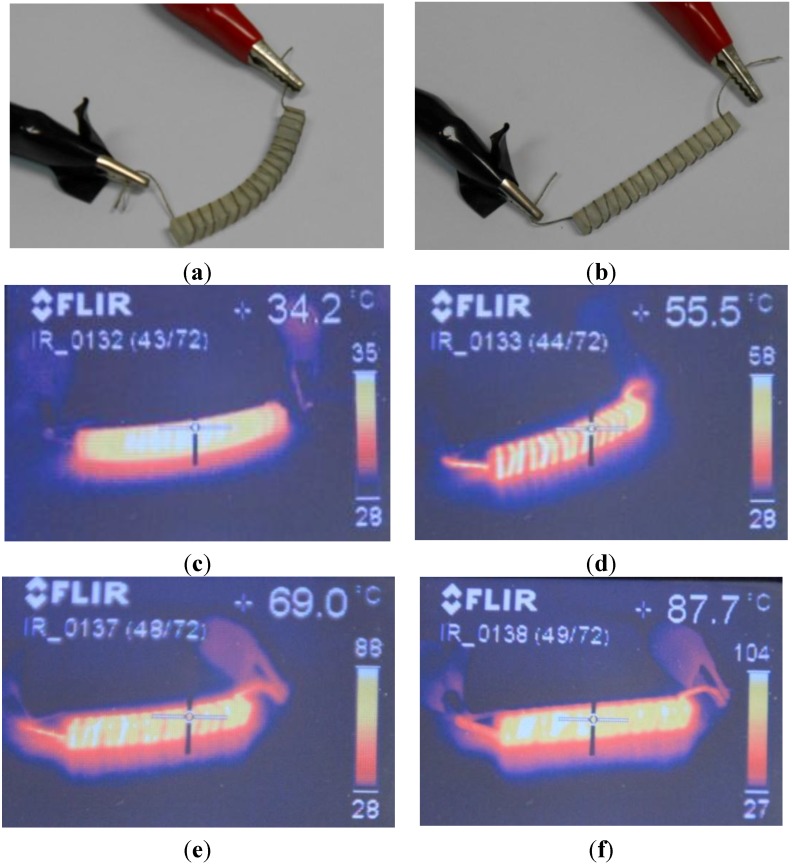
Probe surrounded by conductive thread, with embedded Ag particles, in its temporary shape (bent) and after its original shape recovery through heating (almost complete recovery). Infrared thermographs showing the heating process and the related shape recovery.

Interesting results are also obtained with the conductive thread incorporating Ag nanoparticles, as Figure 4 helps to show by including a probe surrounded by the thread, both in its temporary shape (bended form) and after its original shape recovery through heating (linear form). In this case, as current passes through the whole thread length, the differential resistive paths and related problems, previously detailed for the clearmesh, electrolycra and steel cloth, do not appear. The conductive thread does not mechanically affect the training and recovery processes either.

**Figure 5:**
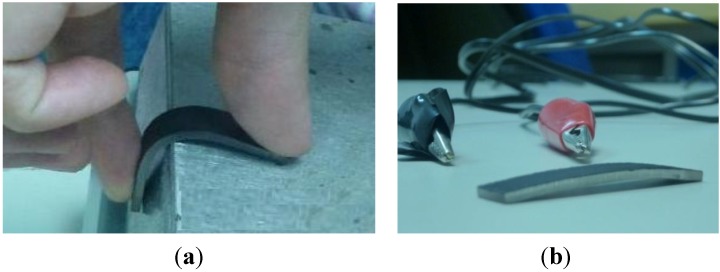

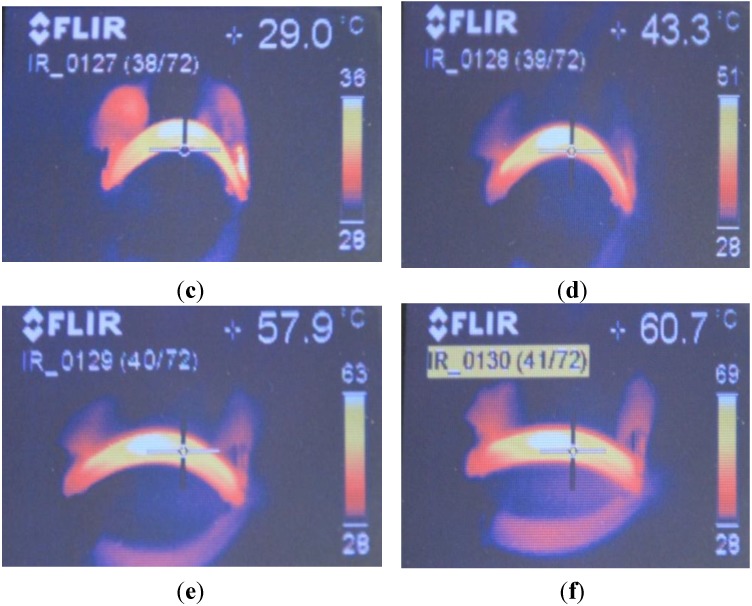
Probe coated with conductive ink in its temporary shape (bended) and after its original shape recovery through heating (almost linear). Infrared thermographs showing the heating process and the related shape recovery, as well as the remarkable homogeneous temperature achieved.

In any case, the most effective and homogeneous results are obtained using the probes coated with the conductive ink including carbon particles, which stands out for its simplicity and flexibility, even though its shape recovery (see Figure 5b) is more limited than when using the conductive thread and the soft-mesh coating. Figure 5 shows a probe coated with conductive ink; Figure 5a in its temporary shape (bent forming 90° between tangents on the extremes), and Figure 5b after its original shape recovery through heating (almost linear), with a recovery ratio above 85%. Solutions based on the conductive thread and on the soft-mesh coating led to values of recovery ratio above 95% (see Table 5 for additional comparison). In spite of the more homogeneous heating processes obtained using the soft mesh, the conductive thread and the conductive ink, especially when comparing these trials with previous devices activated by single or multiple resistors, the temperature variation ranges are greater than expected from the thermal simulations, mainly due to our consideration of perfect contact between the heating element and the shape memory epoxy. However, the simulations helped us importantly for verifying that the different configurations analyzed led to similar results and during the preparation of trials with real prototypes.

**Table 5:** Comparative summary of the typical temperature variation ranges, obtained in shape memory polymeric devices during their activation process, by using different heating strategies. ** We used a recovery ratio *R*_r_ = (θ_t_ − θ_r_)/(θ_t_ − θ_0_); being θ the angle formed among the tangents to the probes on their extremes in the different configurations: θ_0_ before training; θ_t_ after training and θ_r_ after recovery.

Heating element	Typical temperature variation range within the polymer (according to real trials)	Recovery ratio (**)	Reference
Electrotextiles and Faraday film	33–37 °C	>95%	Present study
Conductive thread	25–29 °C	≈100%	Present study
Conductive ink (upon probe bent 90° during training)	18–24 °C	>85%	Present study
Conductive ink (upon pincer opened 90° during training)	17–25 °C	83%	Present study
Conductive ink (upon pincer opened 160° during training)	17–25 °C	76%	Present study
Heating resistors	50–60 °C	>80%	[10,31,32]
Peltier devices	40–50 °C (in the heated zone)	>85%	[19]
Induction heating (coil core)	35–45 °C	>80%	[10]
Induction heating (nanoparticles)	<15 °C	≈100%	[16,20,33]
Light activation (laser heating)	<10 °C (very thin device)	≈100%	[21]

### 3.3. Final Application Example: Shape-Memory Active Catheter End

**Figure 6:**
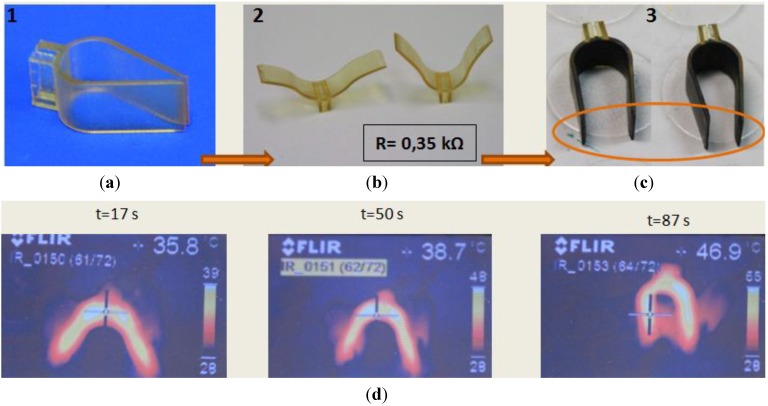
Active shape memory polymer catheter end or pincer. (**a**) Prototype example; (**b**) Training process; (**c**) Geometrical recovery after coating with conductive ink and applying voltage; (**d**) Infrared thermographs showing the heating process and the related shape recovery.

As a final application example, more linked to a potential useful device with a defined three-dimensional geometry, we selected an active shape memory polymer catheter end or surgical pincer. Figure 6 shows some prototypes of the pincer, the training process applied to different copies of the prototype and the geometrical recovery, after coating with conductive ink and applying voltage. We chose the conductive ink, as it provided more interesting results in the previously explained trials with the different probes. The infrared thermographs show the heating process, which is again remarkably homogeneous, and the related shape recovery. Geometrical recovery is also noteworthy, although some residual deformations remain, especially in the pincer which had been more radically trained, probably due to some degree of plastification. Some values about the shape recovery, which may help to decide between the different possibilities, are included in Table 5.

**Figure 7:**
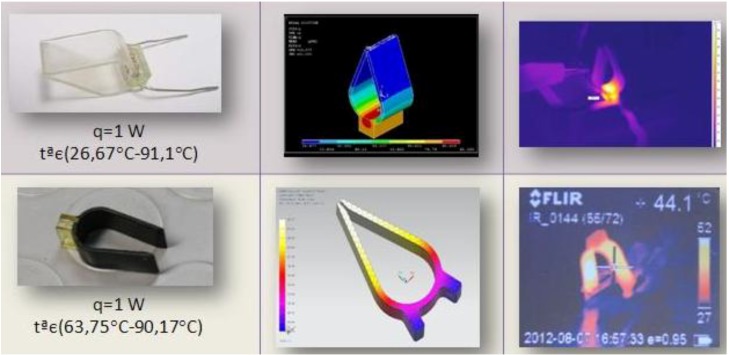
Comparative summary of active catheter ends: One using a resistor as heating element, and one using a coating of conductive ink. Prototypes, simulation results and infrared thermographs help to show that a more homogeneous heating can be achieved when using conductive ink.

Figure 7 includes an interesting comparative summary of an active catheter end, using a resistor as heating element [10,31], and the novel one, using a coating of conductive ink. Prototypes, simulation results and infrared thermographs help to show a more homogeneous heating for the device activated by the conductive ink and support our initial hypothesis.

### 3.4. Final Summary and Discussion

Table 5 includes a quantitative summary of the typical temperature variation ranges, obtained in shape memory polymeric devices during their activation process, by using different heating strategies. The main results from present study are included, together with some data from previous experiences by our team (also measuring with the help of thermographs and the related Thermacam Reporter 8.0 software for increased accuracy, Flyr Systems^®^) and with information taken from groundbreaking research in the field of shape memory polymers.

While in solutions using single or multiple heating resistors, among devices with typical dimensions similar to the ones studied here, typical temperature variation ranges reach values of 50–60 °C during the heating process, the solutions analyzed here led to more homogeneous heating processes [10,31,32], especially those based on the use of conductive thread with Ag nanoparticles and ink with carbon particles. The use of Peltier devices also leads to variation ranges around 40–50 °C in the heated zone, although differences of more than 70 °C between both sides of the Peltier device have been registered, which has proved to be useful for sequential activations [19]. Resistive and inductive heating, using coils embedded into the devices, also leads to important variation ranges [10] and the mechanical influence of the embedded coils can be problematic for the training and recovery stages. However, induction heating is probably the best strategy for a homogeneous heating, when using shape memory polymers with embedded nanoparticles, with the advantage of being contact-less, which can promote the activation of implantable devices from outside the patient’s body [33,34,35]. Although some of the references [20,33] do not provide a quantitative evaluation of temperature differences among the polymer during the heating process, the process is highlighted as homogeneous.

In addition, the excellent review by Leng and colleagues [16] includes interesting thermographs, in which such typical variation ranges, for solutions based on nanoparticles, can be clearly appreciated.

When comparing the proposed low-cost electrotextile or conductive coatings with more advanced solutions based on the use of distributed nanoparticles, it is also important to highlight a limitation of our approach. Embedded nanoparticles can heat the whole volume of the part homogeneously, while the heating textiles and paints discussed here act only on the surface level and can lead to important temperature and even stress gradients through the cross section of the probes, especially if the thickness is higher than 5 mm. In the experiments presented here, the 2 mm thickness of probes and devices helped to limit the cross-sectional temperature gradients below 5 °C, but the mentioned limitation has to be taken into account when using our strategy to activate shape-changes of thicker parts.

Another interesting alternative, for promoting homogeneous and effective heating, is the use of the shape memory polymer as a wave guide for a laser that heats the polymer, typically referred to as light activation [21]. The heating process seems to be also very uniform, although exact quantification is not included and the prototypes shown in the mentioned reference are very thin devices, which may promote uniformity. It will be interesting to follow progress in the different strategies, as well as combinations among them, especially due to the continuously evolving families of shape memory polymers [32,34], to novel approaches enabling tunable multi-shape memory effects [36] and to recent advances in self-healing applications [37,38,39,40], where homogeneous heating is a critical aspect.

## 4. Conclusions

The present study has focused on interesting activation alternatives towards a more homogeneous and effective heating of shape memory polymers. These alternatives are based on coating shape-memory polymers with different kinds of conductive materials, including textiles, conductive threads and conductive paints, which stand out for their easy, secure, rapid and very cheap implementation. Different combinations of shape memory epoxy resin with several coating electrotextiles, conductive films, threads and paints were prepared, simulated with the help of thermal finite element method based resources and characterized using infrared thermography for validating the simulations and overall design process. A final application linked to an active catheter pincer has been detailed and the advantages of using distributed heating instead of punctual heating resistances has been verified and discussed.

Distributed heating and homogeneous surface activation has been achieved in several of the alternatives studied and the technical results, for low-thickness devices, are comparable to those obtained by using advanced shape memory nanocomposites, which have to deal with more complex synthesis, processing and security aspects, all of them having a relevant impact on final device prize. A comparative study of results from different heating strategies, also including information from previous experiences from our team, as well as data from groundbreaking research in the field of shape memory polymers, has been included and discussed. We believe that the materials, methods and results presented may be helpful for researchers in this area and that the solutions presented may be a low-cost alternative, or at least a good complement, to some other relevant strategies aimed at the homogeneous and effective activation of shape memory polymers. In any case, it will be interesting to see how the different activation possibilities evolve and complement each other in the coming years.
